# Metabolomics profiling reveals low blood tyrosine levels as a metabolic feature of newborns from systemic lupus erythematosus pregnancies

**DOI:** 10.3389/fimmu.2024.1335042

**Published:** 2024-01-31

**Authors:** Yao Cai, Zhirong Deng, Qiuping Yang, Guixian Pan, Zao Liang, Ximei Yang, Jie Song, Xin Xiao, Sitao Li

**Affiliations:** ^1^ Department of Pediatrics, The Sixth Affiliated Hospital, Sun Yat-sen University, Guangzhou, China; ^2^ Biomedical Innovation Center, The Sixth Affiliated Hospital, Sun Yat-sen University, Guangzhou, China; ^3^ Department of Pediatrics, The Fifth Affiliated Hospital, Sun Yat-sen University, Zhuhai, China

**Keywords:** systemic lupus erythematosus, metabolomics, tyrosine, newborn, offspring, mass spectrometry

## Abstract

**Introduction:**

Pregnancy outcomes of patients with systemic lupus erythematosus (SLE) have improved over the past four decades, leading to an increased desire for pregnancy among this cohort. However, the offspring of patients with SLE still face the risks of preterm birth, low birth weight, learning disabilities, and neurological disorders, while the causes underlying these risks remain unclear.

**Methods:**

In this study, we analyzed the blood metabolic features of neonates born to 30 SLE patients and 52 healthy control mothers by employing tandem mass spectrometry with the dual aims of identifying the etiology of metabolic features specific to infants born from mothers with SLE and providing new insights into the clinical management of such infants.

**Results:**

We found significant differences in serum metabolite levels between infants born from mothers with SLE and those born from mothers without SLE, including 15 metabolites with reduced serum levels. Further analysis revealed a disrupted tyrosine metabolism pathway in the offspring of mothers with SLE.

**Discussion:**

By constructing a composite model incorporating various factors, such as serum tyrosine levels, gestational age, and birth weight, we were able to accurately differentiate between newborns of SLE and non-SLE pregnancies. Our data reveal significant differences in serum concentrations of amino acids and acylcarnitines in newborns born to mothers with SLE. We conclude that the reduction of blood L-tyrosine levels is a feature that is characteristic of adverse neurological outcomes in infants born from mothers with SLE.

## 1 Introduction

Systemic lupus erythematosus (SLE) is a chronic, multifactorial autoimmune disease that commonly affects women of reproductive age. Pregnancy has not been recommended for patients with SLE in the past because of adverse pregnancy outcomes. Mehta et al. (1, 2) suggested that infants born to mothers presenting with SLE during pregnancy are at risk for various complications, including premature birth, low birth weight, neonatal lupus syndrome, neurodevelopmental disorders, learning disabilities, autism spectrum disorders, and attention deficit hyperactivity disorders (ADHD). However, the specific mechanisms underlying these complications remain unclear. Despite advances in disease monitoring, diagnosis, and the multidisciplinary management of pregnancy (3, 4), patients with SLE still have concerns regarding adverse pregnancy outcomes and the overall future health and development of their offspring (5).

Metabolomics is an emerging omics research method that studies the types, quantities, and dynamic changes in metabolites within the body. It is not only used for disease diagnosis and prognosis assessments, but also serves as an important means of exploring new therapeutic approaches for diseases (6). Among the plethora of metabolomics research methodologies, tandem mass spectrometry (MS/MS) is a fundamental technique that has been extensively utilized in the investigation of newborns and the treatment of inherited metabolic diseases (7–9).

Encouragingly, previous studies have utilized metabolomics to reveal that patients with SLE exhibit significantly reduced levels of certain amino and fatty acids in the circulation (10). Some studies have identified specific blood metabolites as potential biomarkers for the early diagnosis of SLE (11, 12). In contrast, Lee et al. (13) discovered that plasma concentrations of metabolites of phenylalanine and other amino acids were markedly reduced in mid-pregnancy for patients with SLE, and that these changes in the circulating levels of specific metabolites could accurately predict adverse outcomes in pregnancy. These studies have revealed unique blood metabolite variations in pregnant women with SLE and their fetuses that may potentially influence pregnancy outcomes.

The offspring of mothers with SLE are confronted with adverse outcomes, such as preterm birth, low birth weight, neonatal lupus syndrome, and long-term neurological complications whose precise etiology remains elusive (1, 14, 15). Metabolomics investigations have revealed distinct metabolic profiles in both patients and pregnant women with SLE (11, 13). However, it remains unclear whether SLE and the resulting intrauterine environment can lead to alterations in neonatal metabolites and metabolic pathways. Therefore, we investigated the distinctive metabolic features of neonates born to mothers with SLE to analyze the influence of maternal SLE on neonatal blood metabolites and explore potential biomarkers for offspring of mothers with SLE.

## 2 Methods

This study was conducted in accordance with the provisions of the Declaration of Helsinki (revised in October 2013) and was approved by the Ethics Committee of the Sixth Affiliated Hospital of Sun Yat-sen University (ethics Approval Number:2023ZSLYEC-116). The data collected and analyzed in this study were strictly used for research purposes, and detailed patient information was not disclosed.

### 2.1 Study participants

The inclusion criteria for this study were as follows: (1) The experimental group consisted of newborns delivered by mothers with SLE, diagnosed according to the American College of Rheumatology classification criteria for SLE (16). The control group was comprised of newborns born to mothers without SLE. (2) None of the newborns exhibited congenital anomalies or hereditary metabolic disorders. (3) The parents had no prior history of metabolic disorders and no records of alcohol, tobacco, or substance abuse.

The exclusion criteria for our study were as follows: (1) Newborns who, for various reasons, did not undergo blood metabolic testing within 3-7 days after birth, (2) Preterm infants (gestational age less than 259 days) or those requiring intravenous nutrition due to feeding difficulties after birth, and (3) Newborns who developed severe illnesses (such as neonatal sepsis, necrotizing enterocolitis, severe metabolic disorders, and congenital inherited metabolic disorders) or died during hospitalization.

Thirty newborns from pregnancies with SLE (N-SLE group) and 58 newborns from healthy control pregnancies (N-HC group) at the Sixth Affiliated Hospital, Sun Yat-sen University, between January 2017 and December 2021, were included. The demographic characteristics of the two groups of pregnant women are shown in **Table 1**. There were no significant differences in maternal age, pre-pregnancy weight, height, or body mass index between the two groups (*P* > 0.05).

**Table 1:** The demographic characteristics of the two groups of pregnant women.

	SLE (n=30)	HC (n=52)	t/Z	*P*
Age years)	30.3 ± 3.7	28.8 ± 3.5	1.888	0.063
Prenatal weight (kg)	60.9 ± 5.6	63.5 ± 7.8	-1.838	0.070
Height (cm)	158.2 ± 5.4	158.6 ± 5.2	-0.374	0.710
BMI (kg/m^2^)	23.9(22.8, 24.8)	25.3(23.3, 27.2)	-1.733	0.083
TREATMENT		-		
Glucocorticoid (n, %)	25, 83.3%	–		
Hydroxychloroquine (n, %)	26, 86.7%	–		
Cyclosporine (n, %)	8, 26.7%	–		

### 2.2 Sample collection

After the parents had been formally informed and had signed an informed consent form for newborn blood metabolite level testing, peripheral blood samples were collected from the heels of the newborns. Samples were collected after adequate feeding (72 h after birth) using the dried blood spot method on a filter paper. The filter paper was placed on a clean and flat surface, allowed to air-dry for 3-4 hours, and labeled with the bed number and name using a marker pen. The dried filter paper was then sealed in a plastic bag and stored in a -20°C freezer.

### 2.3 Experimental methods

Circular blood spots 3 mm in diameter were prepared from the filter paper using a hole puncher and placed in a 96-well filtration plate. Each well was then filled with 100μL of extraction solution containing the internal standard (methanol: ultrapure water at a ratio of 8:2). The plate was placed in a microplate shaker and shaken at 700 rpm at 45°C for 45 minutes. After centrifugation at 4,000 rpm for 5 minutes(with a centrifuge radius of 4 cm), 75μL of the supernatant was transferred to a 96-well V-bottom plate. The plate was covered with an aluminum film and analyzed using a tandem mass spectrometer (Xevo-TQD, Waters Corp., Milford, MA, USA).

### 2.4 Data analysis

All statistical analyses were performed using SPSS (version 25.0) software, and the results were considered statistically significant using a two-tailed analysis (*P*<0.05). For continuous variables with a normal distribution and homogeneity of variance, the data were described using the mean ± standard deviation format, and between-group comparisons were performed using t-tests. For data that did not follow a normal distribution, the median M (P25, P75) format was used to describe the data, and between-group comparisons were performed using the Mann-Whitney U test. Categorical variables were expressed as numerical values and percentages, and differences between groups were analyzed using the Chi-square test.

MetaboAnalyst 5.0 (https://www.metaboanalyst.ca/) software was used to perform orthogonal partial least squares discriminant analysis (OPLS-DA) multifactorial discriminant analysis to identify metabolites that significantly contributed to the classification. The quality of the OPLS-DA model was ensured by multiple correlation coefficients (R2X and R2Y) and cross-validation (Q2) with 10-fold cross-validation. The statistical significance of serum metabolites with a variable importance plot (VIP) score > 1.0 in the OPLS-DA model was evaluated using t-tests or non-parametric tests. Metabolic pathway analyses and heatmaps were generated using MetaboAnalyst 5.0 software, and scatter plots of differentially expressed metabolites were created using GraphPad Prism 8.0 software. Logistic regression analysis was performed using SPSS 25.0 software to correct for differences in clinical data and the effects of interactions between multiple metabolites, and the best combination model was established. The specificity of the metabolites and combination model was evaluated using receiver operating characteristic (ROC) curve analysis with MedCalc 20.0 software.

## 3 Results

### 3.1 Demographic characteristics

Nilsson et al. (17) have conducted research showing that premature infants possess a specific amino acid and fatty acid metabolome. To minimize the confounding effects of variables specific to premature infants, this study excluded premature infants from the metabolomics analysis. By using our inclusion and exclusion criteria, 30 neonates were included in the experimental group (N-SLE) and 52 neonates were included in the control group (N-HC). The demographic characteristics of the two neonate groups are presented in **Table 2**. There were no significant differences in sex ratio and head circumference between the two groups (*P*>0.05); however, neonates in the SLE group had a lower gestational age, birth weight, and length than those in the control group.

**Table 2:** Characteristics of the study population.

	N-SLE (n=30)	N-HC (n=52)	x^2^/t	*P*
Gender(male/female)	17/13	27/25	0.172	0.678
Gestational age (days)	267.0 ± 5.4	273.3 ± 5.2	-5.153	0.000*
Birth weight (g)	2783.3 ± 354.6	3144.4 ± 303.3	-4.438	0.000*
Length(cm)	48.5 ± 1.8	49.8 ± 1.5	-3.529	0.001*
Head circumference(cm)	32.4 ± 1.2	32.7 ± 1.1	-1.216	0.228

*P<0.05, N-SLE, newborns from pregnancies with systemic lupus erythematosus; N-HC, newborns from healthy control pregnancies.

### 3.2 OPLS−DA model building and score plot

**Figure 1:**
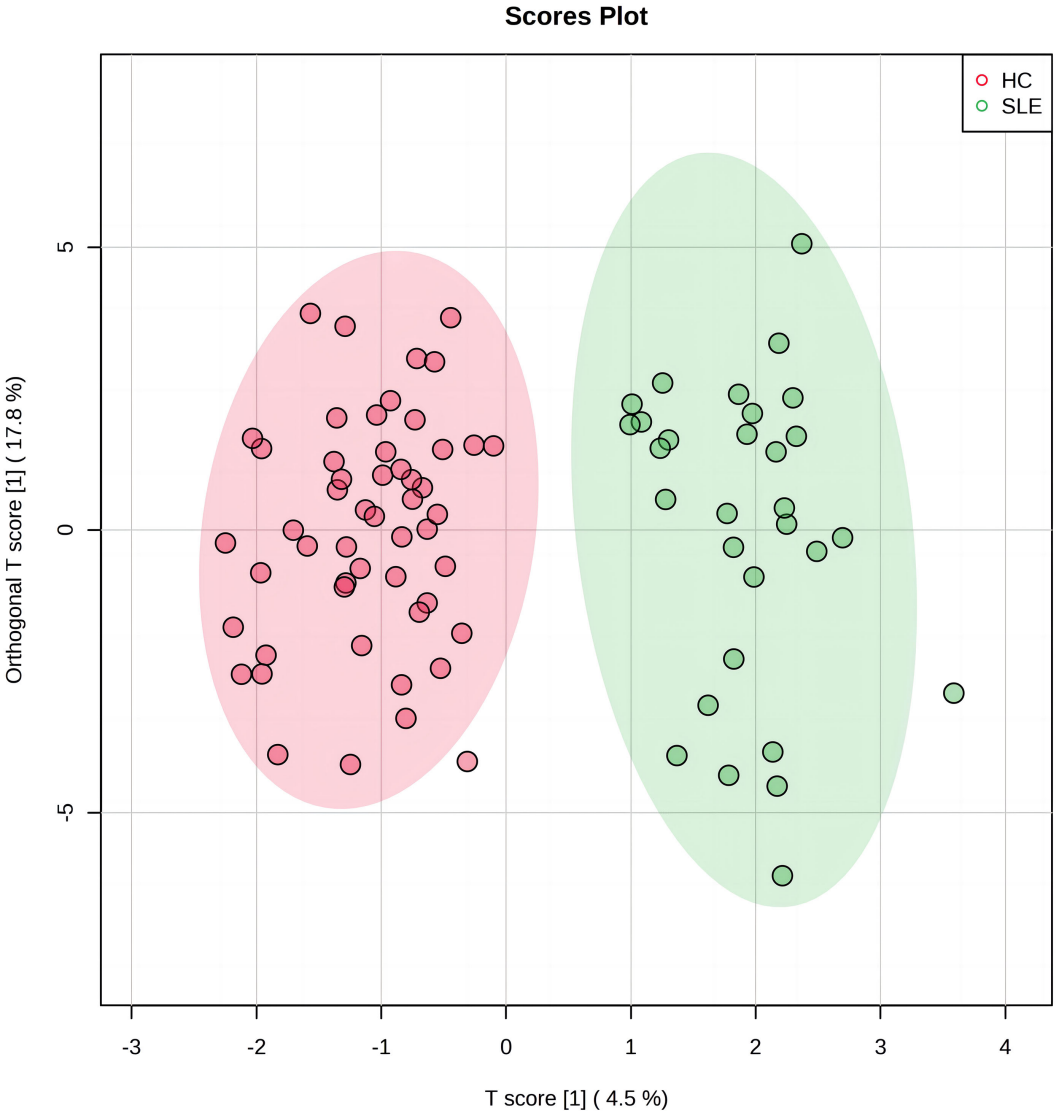
The orthogonal partial least squares discriminant analysis (OPLS-DA) score plot of systemic lupus erythematosus (SLE) group and control group. The horizontal axis represents the predictive component that shows the differences between groups. The vertical axis represents the orthogonal component that shows the within-group dispersion. The green dots on the graph represent the experimental group (n=30), and the red dots represent the control group (n=52).

OPLS-DA was used to analyze the amino acid and acylcarnitine data obtained via blood analysis of the two neonate groups. Our model included three main components: R2X=0.523, R2Y=0.872, and Q2 = 0.594, all exceeding 0.5, indicating that the model had good explanatory power, stability, and predictability, and no overfitting. In **Figure 1**, the experimental group (green dots) and control group (red dots) were significantly separated by OPLS-DA analysis, indicating that there were significant differences in blood metabolite levels between the two groups.

### 3.3 Selection and analysis of differentially expressed blood metabolites

**Figure 2:**
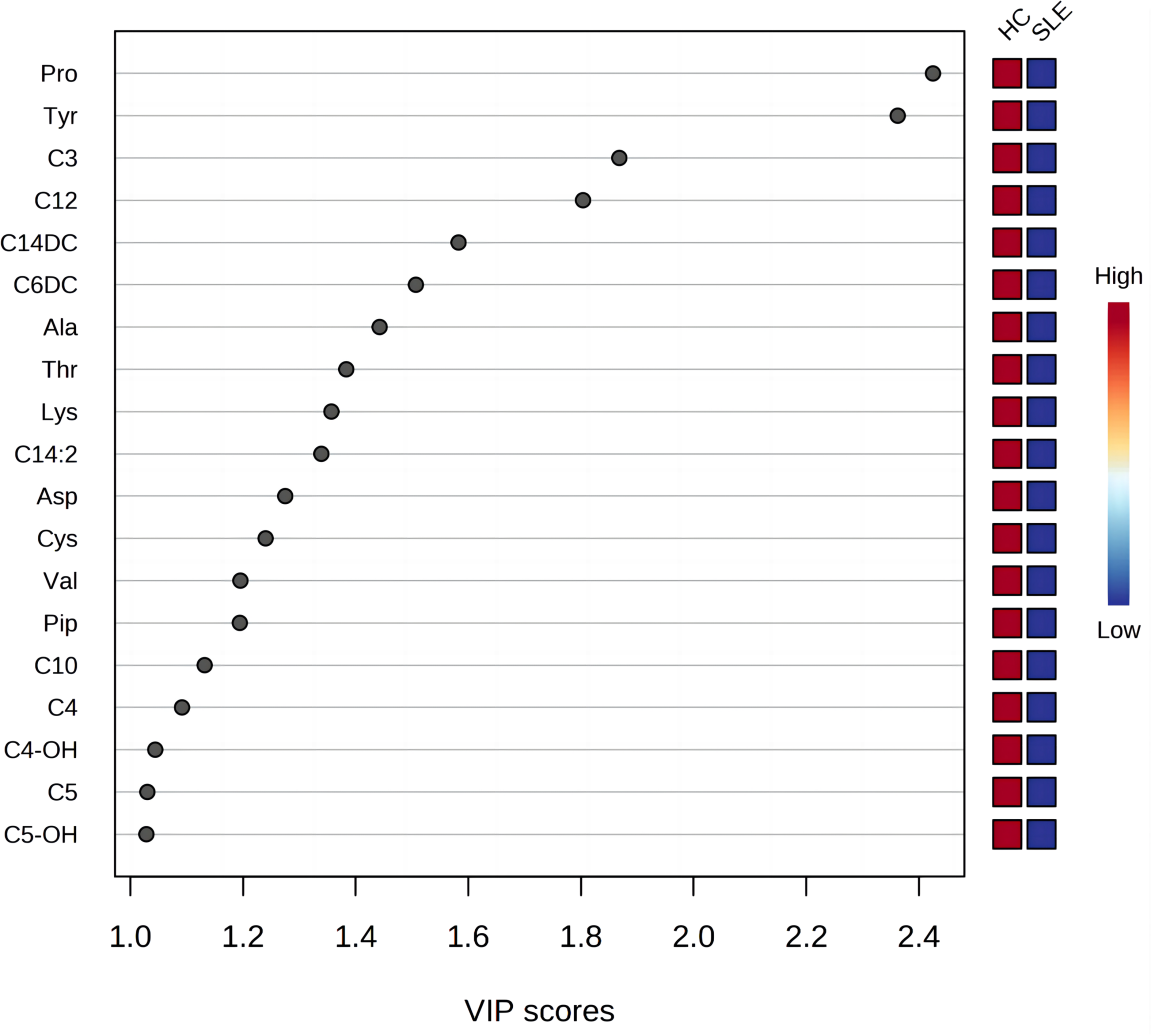
Variable importance for the projection of all blood metabolites. The X-axis represents the variable importance plot (VIP) score (VIP>1 indicates a larger contribution of the metabolite to group discrimination), and the Y-axis represents the compounds. Red and blue colors represent increased and decreased levels of metabolites, respectively.

The most commonly used screening criteria for differentially expressed metabolites were VIP>1 based on OPLS-DA model analysis and significant inter-group differences in individual metabolites (*P*<0.05), as confirmed using multivariate and univariate approaches. Fifteen differentially expressed metabolites were identified (**Figure 2**; **Table 3**).

**Table 3:** Statistical analysis of metabolite levels with a variable importance plot (VIP) score >1.

metabolites	N-HC(n=52)	N-SLE(n=30)	VIP	*P*
Pro^a^	148.92 ± 33.65	110.58 ± 31.79	2.43	0.000
Tyr	65.37(50.13, 83.49)	37.51(31.90, 54.38)	2.36	0.000
C3	1.73(1.32, 2.16)	1.22(0.73, 1.54)	1.87	0.001
C12	0.06(0.05, 0.08)	0.05(0.04, 0.06)	1.80	0.003
C14DC	0.07(0.06, 0.09)	0.06(0.03, 0.07)	1.58	0.004
C6DC	0.02(0.01, 0.02)	0.01(0.01, 0.02)	1.51	0.052
Ala	254.49(217.01, 286.59)	216.69(185.68, 246.96)	1.44	0.006
Thr	152.39(114.54, 187.94)	125.42(99.76, 145.25)	1.38	0.017
Lys^a^	260.90 ± 83.32	210.78 ± 96.48	1.36	0.015
C14:2	0.01(0.01, 0.02)	0.01(0.00, 0.01)	1.34	0.053
Asp	31.28(22.51, 43.04)	24.20(19.66, 30.44)	1.28	0.013
Cys	17.92(15.05, 26.84)	15.26(13.23, 20.91)	1.24	0.043
Val^a^	85.22 ± 22.40	73.76 ± 20.86	1.20	0.025
Pip	12.71(8.83, 17.02)	9.59(6.77, 13.96)	1.19	0.013
C10	0.05(0.04, 0.06)	0.04(0.03, 0.05)	1.13	0.037
C4	0.15(0.12, 0.18)	0.14(0.10, 0.17)	1.09	0.048
C4-OH	0.06(0.05, 0.08)	0.05(0.04, 0.06)	1.04	0.076
C5	0.08(0.06, 0.10)	0.06(0.05, 0.08)	1.03	0.029
C5-OH	0.20(0.15, 0.25)	0.19(0.14, 0.23)	1.03	0.182

For normally distributed data, the t-test was used for between-group comparison, whereas for data that did not follow a normal distribution, the Mann-Whitney U test was used for between-group comparison.

**Figure 3:**
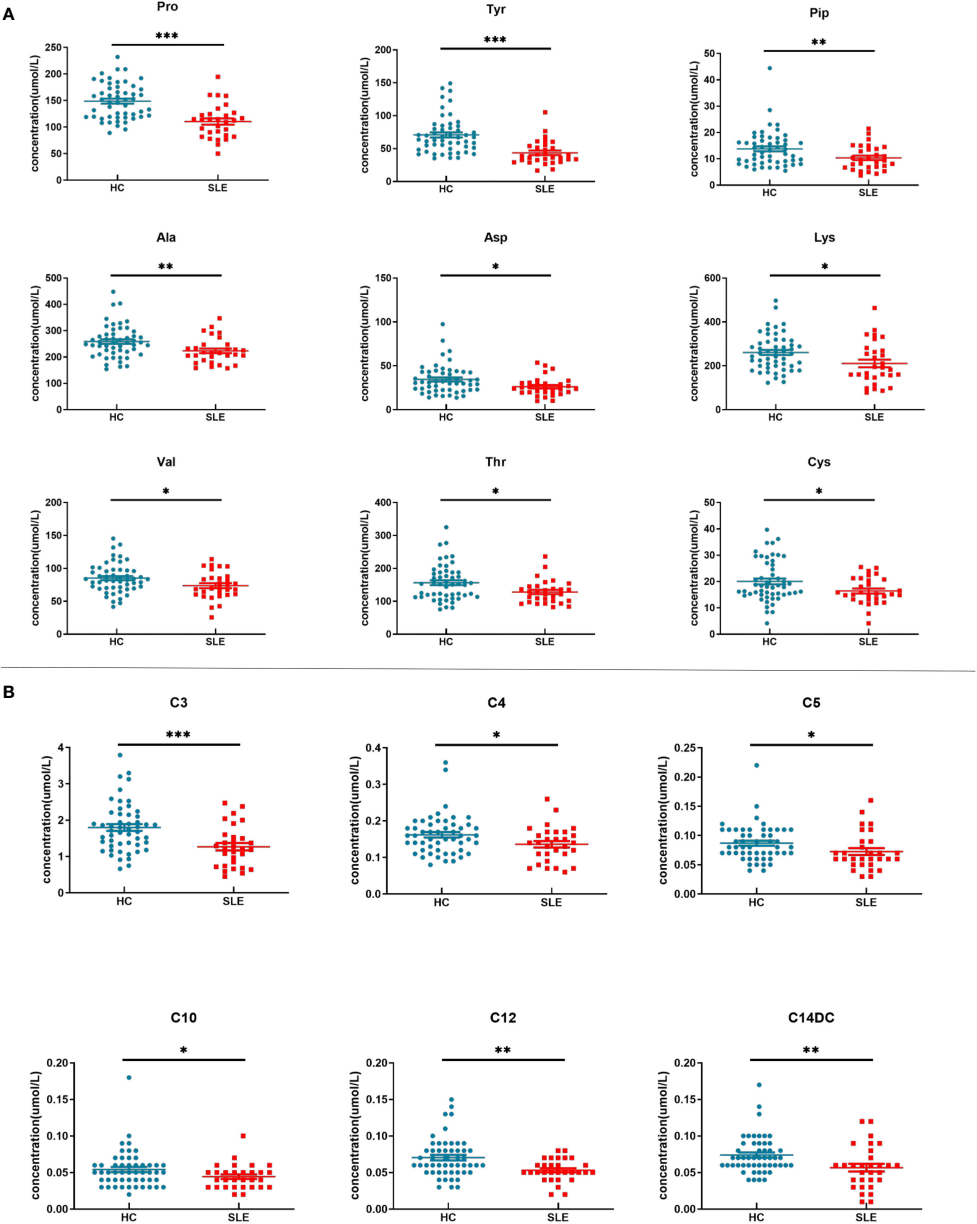
Blood metabolite levels. **(A)** Scatter map of blood amino acid differential metabolites. **(B)** Scatter diagram of hemacylcarnitine differential metabolites. * indicates *P* value between 0.01 to 0.05, ** indicates *P* value between 0.001 to 0.01, and *** indicates *P* value < 0.001.

There were a total of 15 differentially expressed metabolites between the experimental and control neonate groups. A decrease was observed in the levels of the amino acids tyrosine (Tyr), proline (Pro), alanine (Ala), aspartate (Asp), piperidine (Pip), lysine (Lys), valine (Val), threonine (Thr), and cysteine (Cys) in the experimental group compared to the control group (**Figure 3A**). This is similar to the results of metabolomics studies on blood samples from patients with SLE (10). The acylcarnitines propionylcarnitine (C3), butyrylcarnitine (C4), isovalerylcarnitine (C5), dodecanoylcarnitine (C12), O-tetradecanoylcarnitine (C14DC), and decanoylcarnitine (C10) were also decreased in the experimental group compared to the control group (**Figure 3B**).

**Figure 4:**
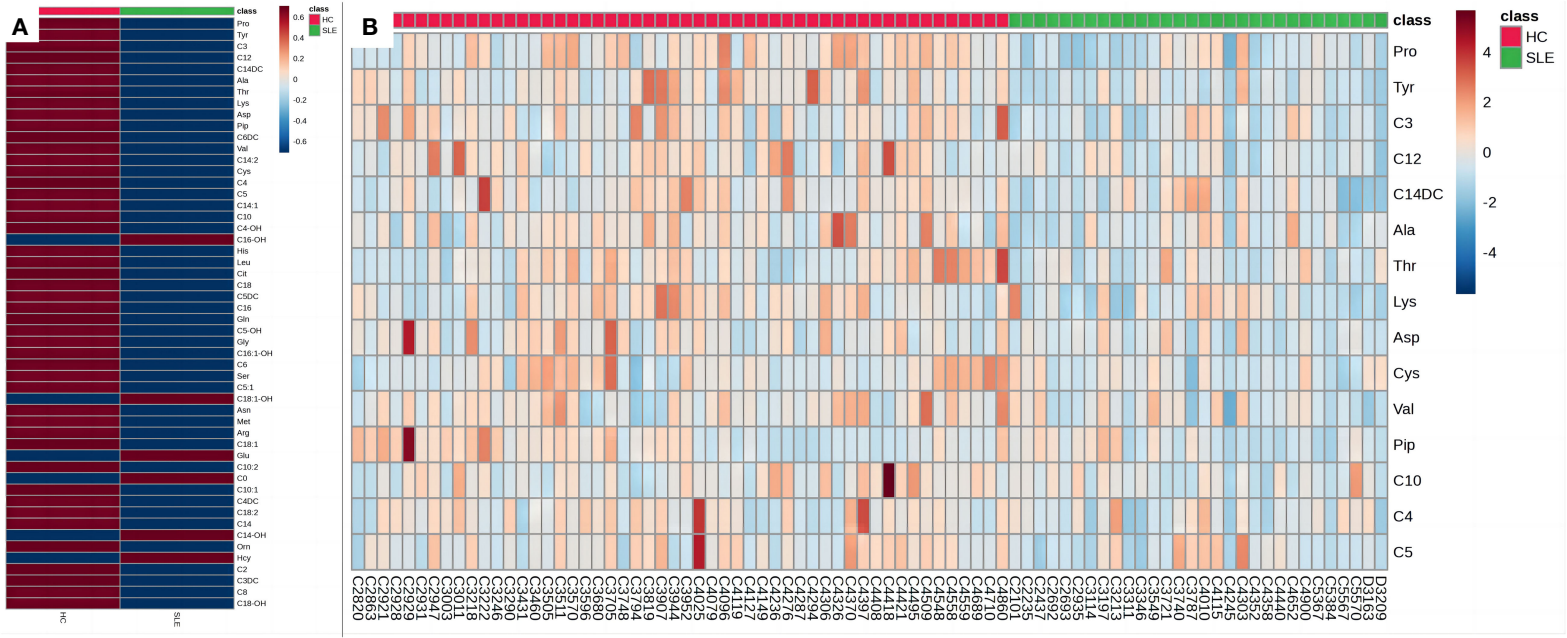
Heat maps of blood metabolites. **(A)** Heat map of blood metabolites (mean values). **(B)** Heat map of differential metabolites. Each grid represents the relative concentrations of different metabolites in a sample. The redder the color, the higher the relative concentration of the metabolite.

To better illustrate the relationship between the samples and the differences in the expression of metabolites in the different samples, we performed a heatmap analysis of the metabolites between the two groups (**Figures 4A, B**). As shown in **Figure 4A**, the levels of most amino acids and acylcarnitines in the experimental group were lower than those in the control group of neonates, suggesting a potential association between energy metabolism deficiency and neonates of mothers with SLE. **Figure 4B** illustrates that the relative concentrations of different metabolites in most samples in the experimental group were lower than those in the control group, revealing a unique blood amino acid and acylcarnitine profile in neonates of mothers with SLE. However, some individual samples, such as C4303, C4010, and C3787, showed higher relative concentrations of certain metabolites that may be related to other postnatal interfering factors or individual differences.

### 3.4 Metabolic pathway analysis

**Figure 5:**
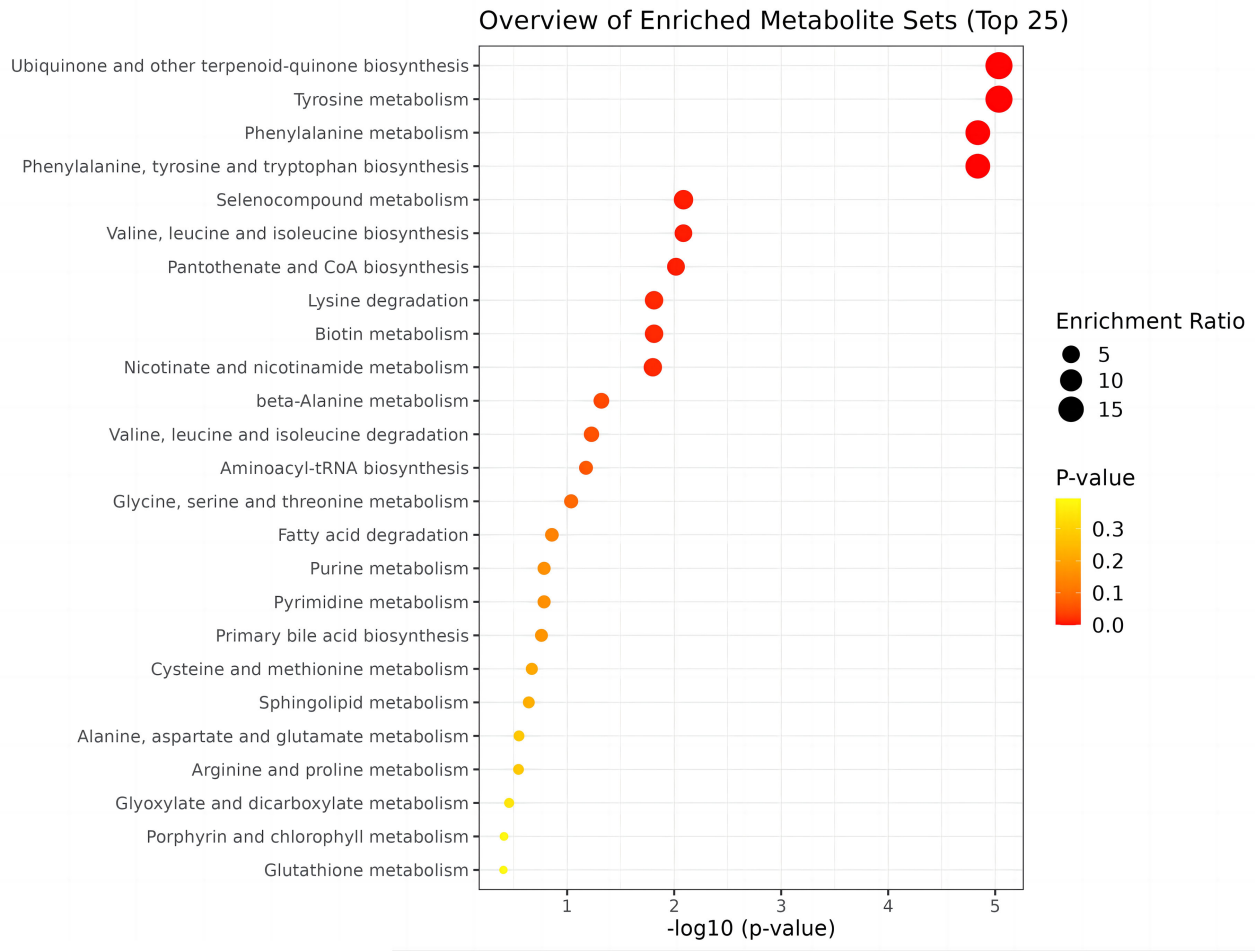
Pathway analysis of metabolite set enrichment analysis.

To identify biologically meaningful patterns based on metabolomics data, pathway analysis was conducted using the Kyoto Encyclopedia of Genes and Genomes (KEGG) metabolic library via MetaboAnalyst 5.0 software. The perturbed metabolic pathways in the blood samples are shown in **Table 4**. Tyrosine and phenylalanine metabolism, as well as phenylalanine, tyrosine, and tryptophan biosynthesis were significantly enriched in the N-SLE group (*P* < 0.01). A dot plot was drawn based on KEGG human metabolic pathways (**Figure 5**). The closer the pathway was to the upper right, the more reliable it was.

**Table 4:** Kyoto Encyclopedia of Genes and Genomes (KEGG) metabolic pathway analysis.

metabolic pathway	Hits/Total Cmpd	*P*	Impact
Tyrosine metabolism	1/42	0.0000092176	0.140
Phenylalanine, tyrosine and tryptophan biosynthesis	2/4	0.00042164	1.000
Phenylalanine metabolism	2/10	0.00042164	0.357
Aminoacyl-tRNA biosynthesis	18/48	0.067	0.167
Ubiquinone and other terpenoid-quinone biosynthesis	1/9	0.0000092176	0.000

**Figure 6:**
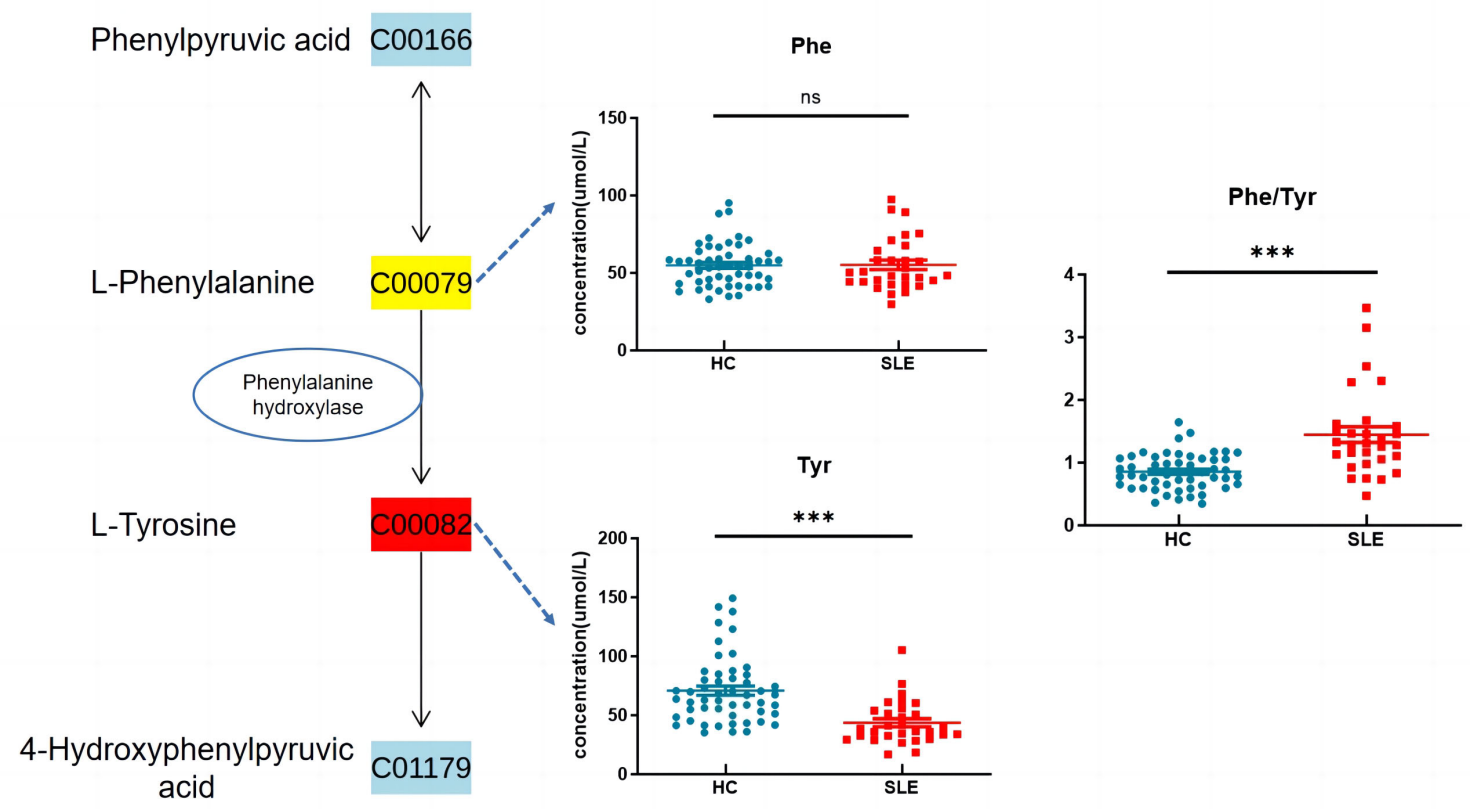
Phenylalanine, tyrosine and tryptophan biosynthesis. ns indicates *P* value > 0.05 , *** indicates *P* value < 0.001.

Based on the KEGG metabolic pathway database, tyrosine is synthesized from phenylalanine (Phe) in the liver through the catalysis of phenylalanine hydroxylase (PAH) in the body. In this study, there was no significant difference in blood phenylalanine levels between the two groups, whereas tyrosine levels were significantly decreased, and the Phe/Tyr ratio was significantly increased in the experimental group (**Figure 6**), suggesting that there may be a decrease in PAH activity in neonates of mothers with SLE, leading to a decrease in tyrosine levels.

### 3.5 Logistic regression and ROC curve analysis

Having established that metabolomics is instrumental in differentiating SLE from normal neonates, we aimed to explore potential biomarkers for SLE.

**Figure 7:**
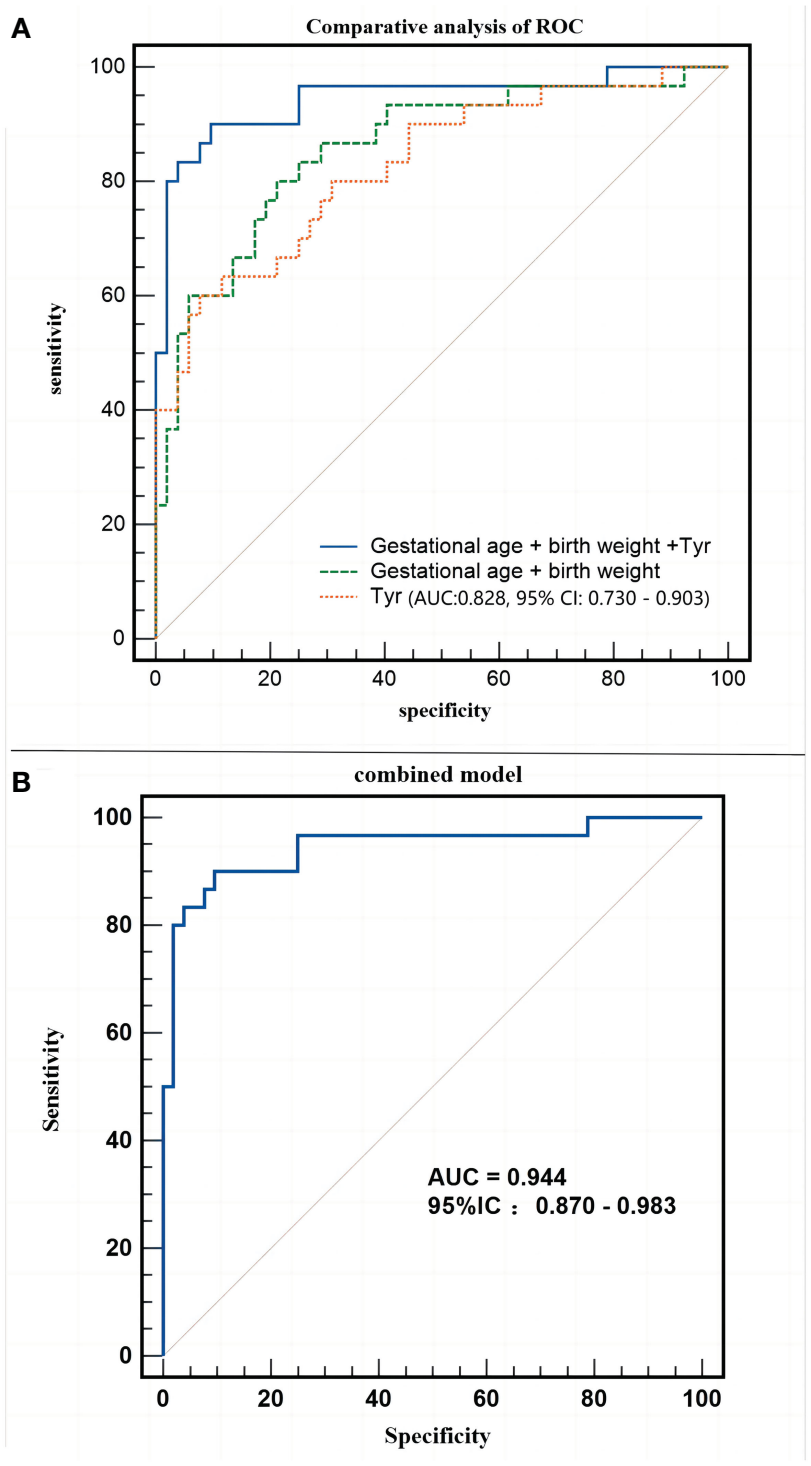
Different models employed in the study. **(A)** Receiver operating characteristic (ROC) curve of combined model (Gestational age + birth weight +Tyr). **(B)** Comparative analysis of ROC of the combined model.

After establishing that metabolomics is instrumental in differentiating neonates with SLE from normal neonates, logistic regression analysis was applied to correct the confounding factors of the clinical characteristics of metabolic products and to explore potential biomarkers for SLE in neonates. Maternal pregnancy age, prenatal weight, height, BMI, neonatal gestational age, birth weight, birth length, and neonatal sex were included in the regression equation to establish a diagnostic model. The regression equation was logit (P) = 85.887 - 0.087 * Tyr - 0.004 * birth weight - 0.256 * gestational age. The combined model could accurately distinguish between experimental and control neonates (AUC:0.944, 95% CI:0.870–0.983, *P* < 0.01, **Figure 7A**), with a sensitivity of 90.00% and specificity of 90.38%.

Does an increase in tyrosine level significantly enhance the ability to distinguish the clinical characteristics of pregnant neonates? **Figure 7B** shows that both blood tyrosine (AUC:0.828, 95% CI:0.730 - 0.903) and clinical characteristics (AUC:0.858, 95% CI:0.763-0.925) have good discrimination efficiency. The comparison of ROC curves suggested that the discriminatory performance of the two was equivalent (Z=0.431, *P*=0.6663), whereas the predictive efficiency of the combination of clinical characteristics and metabolites was significantly better than that of clinical characteristics alone (Z=2.786, *P*=0.0053). This indicates that, apart from changes in gestational age and birth weight, changes in blood tyrosine levels are a unique metabolic feature of the experimental group of neonates.

### 3.6 Association of tyrosine with clinical features

**Figure 8:**
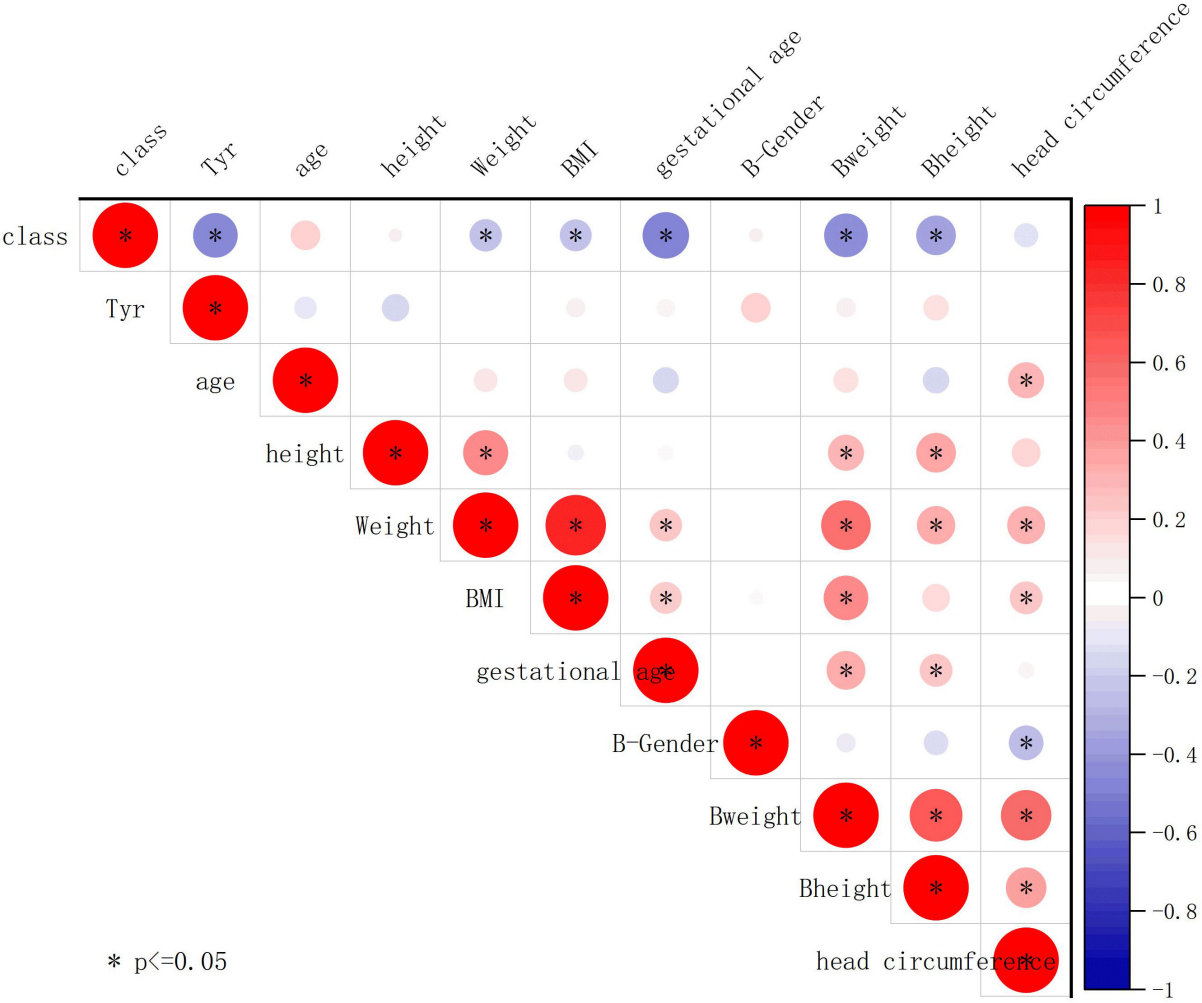
Association of tyrosine with clinical features. The plot displays Pearson correlation coefficients between the contents of different metabolites and clinical parameters. The strength of the correlation is indicated by the color and size of the circles. Positive correlations are represented by red shades, while negative correlations are represented by blue shades. Circles are used to represent statistically significant correlations (P < 0.05).

We further conducted Pearson correlation analysis using Origin 2023 to plot the correlation between tyrosine and clinical characteristics (**Figure 8**). The results showed a negative correlation between tyrosine and class (whether the mother has SLE) (r = -0.47, P < 0.05), while tyrosine had no significant correlation with birth weight (r = 0.091, P > 0.05) or gestational age (r = 0.077, P > 0.05), indicating that tyrosine is minimally affected by these confounding factors.

### 3.7 The impact of maternal drug exposure

**Figure 9:**
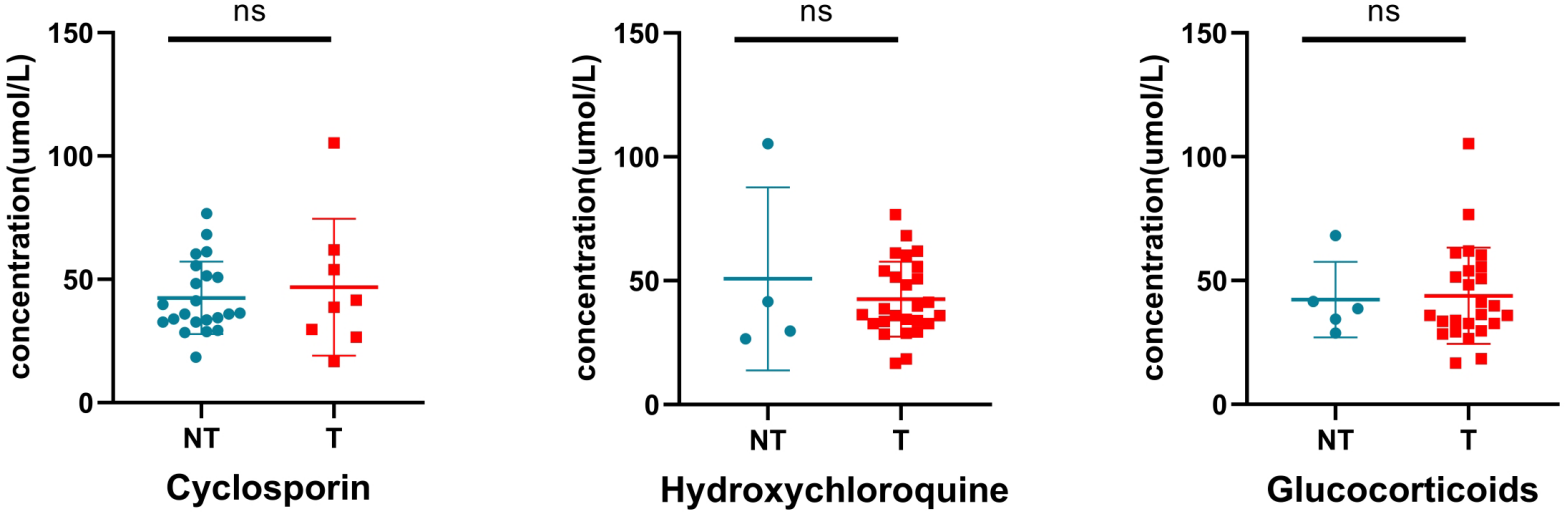
Effect of drug exposure on tyrosine. NT, not treated with specific drugs; T, treated with specific drugs. ns indicates *P* value > 0.05.

Most patients with SLE are administered drugs during pregnancy to control and stabilize the condition of the patient (**Table 1**). We preliminarily assessed the effects of these drugs on blood tyrosine levels. The results showed no significant difference in tyrosine levels between neonates exposed to glucocorticoids, hydroxychloroquine, and cyclosporine and those not exposed to these drugs during pregnancy (**Figure 9**), suggesting that changes in blood tyrosine levels in neonates are not associated with exposure to drugs such as glucocorticoids, hydroxychloroquine, and cyclosporine.

## 4 Discussion

### 4.1 Metabolic spectrum characteristics

To our knowledge, this is the first metabolomics study focused on neonates born to mothers with SLE. We identified 15 differentially expressed metabolites in neonates born to mothers with SLE, including specific profiles of circulating levels of amino acids and acylcarnitines. Among these, the circulating levels of amino acids Tyr, Pro, Ala, Asp, Pip, Lys, Val, Thr, and Cys and of C3, C4, C5, C10, C12, and C14DC acylcarnitines were lower than those in the control group. In contrast, blood tyrosine levels, in combination with gestational age and birth weight, could better distinguish neonates born to mothers with SLE, with an AUC value of 0.944 for the combined predictive model, indicating robust discriminatory power.

Interestingly, our study found a significant decrease in cysteine levels in newborns of SLE pregnancies. Perl et al. have reported that the depletion of cysteine has been linked to the accumulation of kynurenine as well as mTOR activation in SLE (18). Due to funding limitations, we did not measure levels of kynurenine in this study. Further research is needed to investigate whether the depletion of cysteine and the accumulation of kynurenine in SLE mothers may affect newborn metabolism.

Reduced levels of some amino acids and acylcarnitines in neonates born to mothers with SLE may be related to their lower gestational ages and birth weights. Yang et al. (19) found that even with the influence of maternal nutrition, the circulating levels of many amino acid were significantly decreased in preterm and low birth weight infants compared to normal term infants, suggesting the importance of timely amino acid supplementation for these infants. Ivorra et al. (20) found through blood metabolomics that the levels of plasma proline, glutamine, and alanine were decreased in low birth weight infants compared to normal weight infants. Li Sitao et al. (21) found through urine metabolomics analysis that some amino acids (such as valine, lysine, and leucine) and acylcarnitines were decreased in extremely low birth weight infants. Enrichment analysis of metabolic pathways suggested a significant downregulation of the aminoacyl-tRNA biosynthesis pathway, indicating a negative nitrogen balance in neonates that is not conducive to growth and development. These studies suggest that neonates born to mothers with SLE may have higher energy demands due to their lower gestational age and birth weight but may also have insufficient energy due to impaired fatty acid utilization, leading to complications such as hypothermia, hypoglycemia, and neonatal hyperbilirubinemia. This highlights the importance of adequate nutrient supplementation in neonates born to mothers with SLE during early life.

This study found a general decrease in circulating amino acid and acylcarnitine levels in neonates born to mothers with SLE. Logistic regression analysis showed that, except for blood tyrosine levels, changes in other metabolites were mainly enriched in the aminoacyl-tRNA biosynthesis metabolic pathway that may be due to the lower gestational age and birth weight of neonates born to mothers with SLE.

### 4.2 The decrease in L-tyrosine is a characteristic change

Tyrosine (Tyr or L-tyrosine) is a ketogenic and glucogenic amino acid and is one of the 20 amino acids used in protein biosynthesis. Tyrosine can be converted in the human body into important compounds such as dopamine, norepinephrine, epinephrine, and thyroid hormones (22) and is closely related to the functioning of the nervous system (23).

Studies on tyrosine depletion have shown that when participants consume an amino acid mixture that does not contain tyrosine and its precursor, phenylalanine, a sharp decrease in tyrosine and catecholamine levels leads to decreased behavioral motivation (24) or cognitive impairment (25). Consistent with these findings, Hase et al. (26) found that adequate tyrosine supplementation can significantly improve mental health not only by enhancing memory and information processing efficiency but also by alleviating emotional stress. Jongkees et al. (27) suggested that tyrosine supplements are effective cognitive enhancers when neurotransmitter function is intact, and dopamine or norepinephrine is temporarily depleted.

Our study found that after adjusting for confounding factors, such as gestational age and birth weight, blood tyrosine levels remained significantly lower in neonates born to mothers with SLE. The main sources of blood tyrosine levels are the digestion and absorption of food and the biosynthesis of phenylalanine. Zhang et al. (28) found through metabolomics analysis that tyrosine, proline, and asparagine were decreased in serum but significantly increased in feces, suggesting that patients with SLE may have impaired intestinal function, leading to intestinal permeability damage and insufficient amino acid absorption. Another study with similar conclusions found that the ratio of phenylalanine to tyrosine increased in patients with trauma and sepsis and during chronic immune activation or inflammation (29). Julian et al. (30) found through protein structure analysis that this may be related to the decreased activity of phenylalanine hydroxylase under oxidative stress.

Therefore, we speculate that maternal-derived antibodies entering the neonate body activate oxidative stress, leading to impaired intestinal function and decreased phenylalanine hydroxylase activity that, in turn, leads to a significant decrease in blood tyrosine levels. In addition, current family clustering and monozygotic twin studies suggest that SLE has significant genetic susceptibility; however, current genetic analyses can only explain a small part of this susceptibility (31). The metabolomics of neonates are relatively less influenced by factors and have been successfully used for screening congenital defects. This may provide supplementary evidence for the study of genetic factors (32). Similarly, there are low levels of blood tyrosine in patients with SLE, and we need to observe whether the decreased tyrosine levels in the serum of neonates born to mothers with SLE persist in subsequent follow-up studies and explore whether there is a decrease in tyrosine levels in the population with early onset SLE.

Unfortunately, there are relatively few studies on the prognosis of offspring born to mothers with SLE, with most focusing on neurological disorders, including neurodevelopmental disorders, learning and speech disorders, ADHD, and autism spectrum disorders. Most studies have suggested an association between offspring of mothers with SLE and long-term neurological disorders (33–36). Fjodor et al. (33) studied the neurodevelopment of offspring of women with SLE and found that 21.4%–24.6% of this cohort had a higher incidence of learning disabilities than the general population. In addition, studies by Vinet et al. (34, 35) found that attention deficit disorders and autism spectrum disorders were more common in offspring of SLE mothers, with a risk two to three times higher than that in the general population.

The reasons for long-term neurological disorders in offspring of SLE mothers are not clear; however, according to Lee et al. (37), they may be related to inflammation mediated by antibodies of maternal origin. Vumma et al. (38) found that pro-inflammatory cytokines (such as interleukin-1β, IL-6) and oxidative stress reduced the uptake of tyrosine in human fibroblasts, and it is speculated that the long-term chronic inflammation and stress in autoimmune diseases may cause abnormal transport of tyrosine, leading to dopamine and norepinephrine neurotransmission disorders in the brain. Based on this, we speculate that oxidative stress caused by maternal autoimmune antibodies reduces the absorption and synthesis of tyrosine, leading to a decrease in tyrosine, dopamine, and norepinephrine levels in the brain that increases the susceptibility of offspring of mothers with SLE to neurological and psychiatric disorders. However, further verification and validation of these findings are required.

### 4.3 Summary and limitations

Collectively, we found that blood tyrosine levels were significantly reduced at the metabolite level in newborns of mothers with SLE. After logistic regression correction for confounding factors, such as gestational age and birth weight, tyrosine was the only specific blood metabolite to be identified. To our knowledge, this is the first study to explore the impact of mothers with SLE on their newborns using metabolomics. Low blood tyrosine levels may be associated with adverse outcomes such as learning disabilities, autism spectrum disorders, and delayed neurological development in offspring of mothers with SLE. This study may lead to better nutritional supplementation recommendations for correcting metabolic disorders and promoting growth and development. However, this study has some limitations, and further research is needed to confirm these findings.

This is a retrospective study that lacks completely comparable clinical data and follow-up content and has not been able to verify the effect of blood tyrosine levels or explore in depth the impact of blood tyrosine levels on offspring of mothers with SLE. How low blood tyrosine levels arise in offspring of mothers with SLE, the relationship between low blood tyrosine levels and the neurological and psychiatric systems observed in these offspring involves multiple factors such as autoimmune disorders and amino acid regulation of the nervous system that requires more extensive and comprehensive research to further clarify the complex regulatory mechanisms involved.

## Data availability statement

The raw data supporting the conclusions of this article will be made available by the authors, without undue reservation.

## Ethics statement

The studies involving humans were approved by Medical Ethics Committee of the Sixth Affiliated Hospital of Sun Yat-sen University. The studies were conducted in accordance with the local legislation and institutional requirements. Written informed consent for participation in this study was provided by the participants’ legal guardians/next of kin. Written informed consent was obtained from the individual(s), and minor(s)’ legal guardian/next of kin, for the publication of any potentially identifiable images or data included in this article.

## Author contributions

YC: Conceptualization, Funding acquisition, Writing – original draft. ZD: Data curation, Software, Writing – original draft. QY: Investigation, Writing – original draft. GP: Investigation, Writing – original draft. ZL: Writing – original draft, Investigation. XY: Writing – original draft, Investigation. JS: Methodology, Writing – review & editing. XX: Conceptualization, Project administration, Writing – review & editing. SL: Conceptualization, Funding acquisition, Writing – review & editing.
